# Organochlorine pesticides and risk of papillary thyroid cancer in U.S. military personnel: a nested case-control study

**DOI:** 10.1186/s12940-024-01068-0

**Published:** 2024-03-19

**Authors:** Jennifer A. Rusiecki, Jordan McAdam, Hristina Denic-Roberts, Andreas Sjodin, Mark Davis, Richard Jones, Thanh D. Hoang, Mary H. Ward, Shuangge Ma, Yawei Zhang

**Affiliations:** 1 Department of Preventive Medicine and Biostatistics, Uniformed Services University of the Health Sciences, 4301 Jones Bridge Road, Room E-2009, Bethesda, MD 20814 USA; 2 Murtha Cancer Center Research Program, 4494 North Palmer Road, Bethesda, MD USA; 3 Henry M. Jackson Foundation for the Advancement of Military Medicine, 1401 Rockville Pike, Rockville, MD USA; 4 Oak Ridge Institute for Science and Education (ORISE), Oak Ridge, TN USA; 5 Division of Laboratory Sciences, Centers for Disease Control and Prevention (CDC), Atlanta, GA USA; 6 Division of Endocrinology, Department of Medicine, Uniformed Services University of the Health Sciences, Bethesda, MD USA; 7 Division of Cancer Epidemiology and Genetics, National Cancer Institute, Rockville, MD USA; 8 Department of Biostatistics, Yale School of Public Health, New Haven, CT USA; 9 Department of Environmental Health Sciences, Yale School of Public Health, New Haven, CT USA; 10 Department of Cancer Prevention and Control, National Cancer Center/National Clinical Research Center for Cancer/Cancer Hospital, Chinese Academy of Medical Sciences and Peking Union Medical College, Beijing, China

**Keywords:** Organochlorine pesticides, Papillary thyroid cancer, Hexachlorobenzene, Hexachlorocyclohexane

## Abstract

**Background:**

The effects of organochlorine pesticide (OCP) exposure on the development of human papillary thyroid cancer (PTC) are not well understood. A nested case-control study was conducted with data from the U.S. Department of Defense Serum Repository (DoDSR) cohort between 2000 and 2013 to assess associations of individual OCPs serum concentrations with PTC risk.

**Methods:**

This study included 742 histologically confirmed PTC cases (341 females, 401 males) and 742 individually-matched controls with pre-diagnostic serum samples selected from the DoDSR. Associations between categories of lipid-corrected serum concentrations of seven OCPs and PTC risk were evaluated for classical PTC and follicular PTC using conditional logistic regression, adjusted for body mass index category and military branch to compute odds ratios (OR) and 95% confidence intervals (CIs). Effect modification by sex, birth cohort, and race was examined.

**Results:**

There was no evidence of associations between most of the OCPs and PTC, overall or stratified by histological subtype. Overall, there was no evidence of an association between hexachlorobenzene (HCB) and PTC, but stratified by histological subtype HCB was associated with significantly increased risk of classical PTC (third tertile above the limit of detection (LOD) vs. <LOD, OR = 1.61, 95% CI, 1.09, 2.38; *p* for trend = 0.05) and significantly decreased risk of follicular variant PTC (third tertile above the limit of detection (LOD) vs. <LOD, OR = 0.38, 95% CI, 0.16, 0.91; *p* for trend = 0.04). Further stratified by sex, risk of classical PTC was higher for females (third tertile above LOD vs. <LOD, OR = 2.23, 95% CI: 1.23, 4.06; *p*-trend = 0.02) than for males (OR = 1.22, 95%CI: 0.72–2.08; *p*-trend = 0.56), though the test for interaction by sex was not statistically significant (*p*-interaction = 0.30). Similarly, β-hexachlorocyclohexane (β-HCCH) was associated with a higher risk for classical PTC for women with concentrations ≥LOD versus <LOD (OR = 1.76, 95% CI: 1.07, 2.89), while the effects were null for men. There were no consistent trends when stratified by race or birth year.

**Conclusions:**

The U.S. Environmental Protection Agency has classified HCB and other OCPs we studied here as probable human carcinogens. Our findings of increased risks for classical PTC associated with increased concentrations of HCB and β-HCCH, which were stronger among females, should be replicated in future studies of other populations.

**Supplementary Information:**

The online version contains supplementary material available at 10.1186/s12940-024-01068-0.

## Background

Environmental exposures have been implicated as contributing to the rising incidence rates of thyroid cancer, which have been increasing faster than those of any other malignancy in the United States and worldwide [11, 34, 38], though over the last decade (i.e., since 2013) incidence rates have appeared to level off in the United States according to United States Cancer Statistics [76]. Most of the increase in this endocrine malignancy has been for papillary thyroid cancer (PTC), the most common histological type of thyroid cancer [85, 86]. This increase has been especially notable in females, with the incidence rate of PTC nearly three-times higher than in males [63]. PTC incidence has been reported to be higher in the U.S. military than in the general U.S. population among White females, Black females, and Black males [16]. Despite rising incidence rates of thyroid cancer for several decades, there are gaps in our understanding of the etiology of thyroid cancer in humans. Improvements in detection and diagnostics only partially explain the increase in thyroid cancer over past decades [38, 77, 82]. Established risk factors include radiation exposure, family history, improper iodine intake, and obesity [44], but endocrine disrupting chemical (EDC) exposure has also been suggested as a risk factor because these chemicals may alter thyroid function and have been associated with various cancers [1, 9, 74, 83, 89].

Organochlorine pesticides (OCPs) are a class of synthetic chlorinated hydrocarbon EDCs used widely from the 1940s until the early 1970s, when many of them were banned in the United States [78]. Despite being banned, OCPs are persistent in the environment, some of them being generated inadvertently as byproducts or contaminants in the manufacture of other chlorinated chemicals [65], and they can remain in human tissue for many years after initial exposure. The U.S. Environmental Protection Agency (EPA) classifies several OCPs as probable human carcinogens (Group B2), including hexachlorobenzene (HCB), hexachlorocyclohexane (HCCH), dichlorodiphenyltrichloroethane (DDT) and its primary metabolite, dichlorodiphenyldichloroethylene (DDE), and chlordane [80]. Animal studies by which the EPA has classified the carcinogenicity potential of these OCPs have reported thyroid cancer from exposure to HCB [18] and DDE [17]. Similarly, the International Agency for Research on Cancer (IARC) classifies HCB, HCCHs, chlordane, and mirex as *possibly carcinogenic to humans* (Group 2B) and DDT as *probably carcinogenic to humans* (Group 2A) [27–29]. Animal studies by which the IARC has classified the carcinogenicity potential of these OCPs have reported thyroid cancer from exposure to HCB [30].

The small body of literature investigating the association between OCP exposures and thyroid cancer in humans has presented mixed results. Past studies have reported inverse associations between serum concentrations of DDT metabolites and thyroid cancer, with null findings among other OCPs [42], increased risk of thyroid cancer with self-reported exposure to lindane, an HCCH isomer, among male pesticide applicators [41], and null findings between self-reported insecticide use and thyroid cancer risk in female spouses of insecticide applicators [45]. In a case-control study based in Connecticut (USA) including 250 female incident PTC cases and 250 controls, no significant associations were found between serum concentrations of any OCPs and PTC [15]. Another study conducted in the village of Flix, Spain detected unusually high concentrations of HCB in the air and sera of volunteers and observed an excess of incident thyroid cancer in males, but not females [21]; however, this report included a small sample size and the 95% confidence intervals (CIs) were wide.

One study of residents on St. Lawrence Island, Alaska (USA), the location of two formerly used defense sites (FUDS), observed statistically significant increases in serum HCB concentrations and elevated but non-significant concentrations of sum-chlordane and sum-DDT among residents closest to a FUDS compared to residents farthest from the site when controlling for age and sex [7], suggesting military sites may be point sources of OCP exposure. Additionally, the EPA’s Superfund program is responsible for cleaning up some of the most contaminated land in the US. Over 100 Department of Defense (DoD) sites that have been designated as Superfund sites have listed OCPs (i.e., chlordane, *p’p*,-DDE, *p’p*,-DDT, HCB, β-HCCH, mirex, and/or trans-nonachlor) as contaminants of concern (https://cumulis.epa.gov/supercpad/cursites/srchsites.cfm, accessed 13 June 2023), or substances found at the site that the EPA has determined pose an unacceptable risk to human health or the environment. Reports also indicate potentially high concentrations of chlordane in and around military housing due to its use as a termite control agent until it was banned in 1988 [79].

Because of the inconclusive findings related to exposure to OCPs and thyroid cancer and the potential for exposure to OCPs in both the general population and the military, and given that incidence of thyroid cancer has increased substantially in both the general U.S. population and U.S. military personnel, particularly in females [16, 23], we conducted a nested case-control study using data from the DoD Automated Central Tumor Registry (ACTUR) and the Defense Medical Surveillance System (DMSS). Together with pre-diagnostic serum samples from the Department of Defense Serum Repository (DoDSR), we investigated the associations of PTC with serum concentrations of OCPs.

## Methods

### Study population

The study population for our nested case-control study has been previously described [25, 26]. Briefly, 742 case-control pairs were selected from 3.9 million U.S. military service members whose serum samples, leftover from routine human immunodeficiency virus antibody testing and from collections before and after deployment, were stored in the DoDSR and who met the same inclusion criteria as applied here for cases and controls [57, 58, 67]. Incident PTC cases were identified via the DoD ACTUR using International Classification of Diseases for Oncology, 3rd edition (ICD-O-3) histology codes: 8050, 8260, and 8340–8343 and queried between the years of 2000 and 2013. Two of the most common histological subtypes of PTC are classical and follicular variants. The classical variant is characterized by the presence of true papillae with a fibrovascular core and the nuclear features of papillary carcinoma [84]. The follicular variant is characterized as a tumor with both the nuclear features typical of PTC and a follicular growth pattern [22]. Most cases (80.9%) were diagnosed with the classical PTC histological subtype (Table 1). Histologically confirmed PTC cases also met the following inclusion criteria: 1) having at least three 0.5 mL pre-diagnostic serum samples and one 0.5 mL post-diagnostic serum sample stored in the DoDSR since 1989, 2) PTC diagnosis between 2000 and 2013, and 3) at least 21 years of age at PTC diagnosis. PTC cases with a history of any other cancers, except for non-melanoma skin cancer, before the date of thyroid cancer diagnosis were excluded from our study. Controls with no history of any cancer diagnosis, except for non-melanoma skin cancer, were randomly selected from the DoDSR and individually matched (i.e., one-to-one) to cases on date of birth (± 1 year), sex, race/ethnicity, military component at diagnosis/matching (active duty/reserve), and midpoint of dates of the selected four samples drawn (± 1 year). Demographic and military characteristics for all cases and controls (i.e., birth year, sex, race/ethnicity, serum collection dates, height, and weight at the time of military accession, and military service branch) were obtained from the DMSS. All study procedures were reviewed and approved by the Uniformed Services University Institutional Review Board, the Walter Reed National Military Medical Center, the DoD Joint Pathology Center, the Human Investigation Committee of Yale University, and the Centers for Disease Control and Prevention (CDC). The Uniformed Services University Institutional Review Board deemed the study to be exempt. The CDC laboratory’s involvement measuring deidentified human specimens did not constitute engagement in human-subjects research.

**Table 1:** Characteristics of the study population

Characteristic	Cases (*N* = 742)		Controls (*N* = 742)		*P*-value ^1^
	N	(%)	N	(%)	
Age at serum sample collection (yr)					
17–19	123	(16.6)	121	(16.3)	
20–29	349	(47.0)	352	(47.4)	
30–39	220	(29.7)	221	(29.8)	
40–52	50	(6.7)	48	(6.5)	0.99
Mean ± SD	27.3	± 7.4	27.2	± 7.3	
Age at PTC diagnosis (yr) ^a^					
20–29	210	(28.3)	203	(27.4)	
30–39	310	(41.8)	323	(43.5)	
40–49	185	(24.9)	179	(24.1)	
50–62	37	(5.0)	37	(5.0)	0.92
Mean ± SD	35.1	± 8.1	35.1	± 8.0	
Sex					
Female	341	(46.0)	341	(46.0)	
Male	401	(54.0)	401	(54.0)	> 0.99
Race/ethnicity					
Non-Hispanic White	468	(63.1)	467	(62.9)	
Non-Hispanic Black	131	(17.6)	132	(17.8)	
Hispanic	68	(9.2)	68	(9.2)	
Other	55	(7.4)	55	(7.4)	
Unknown	20	(2.7)	20	(2.7)	> 0.99
BMI (kg/m2)					
16.3–24.9	257	(34.6)	285	(38.4)	
25-34.8	164	(22.1)	139	(18.7)	
missing	321	(43.3)	318	(42.9)	0.17
Service branch					
Army	299	(40.3)	253	(34.1)	
Air Force	193	(26.0)	150	(20.2)	
Navy	186	(25.1)	248	(33.4)	
Marines & Coast Guard	64	(8.6)	91	(12.3)	**< 0.0001**
Years from serum sample collection to PTC diagnosis ^b^					
3–4	115	(15.5)	111	(14.9)	
5–9	345	(46.5)	350	(47.2)	
10–12	282	(38.0)	281	(37.9)	0.95
Mean ± SD	8.4	± 2.7	8.4	± 2.7	
Tumor size (mm)					
≤10	235	(31.7)			
>10	463	(62.4)			
Missing	44	(5.9)			
Histological type					
Classical PTC	600	(80.9)			
Follicular variant of PTC	142	(19.1)			

Bolding denotes statistical significance (*p*<0.05)

*Abbreviations:* *BMI* Body mass index, *PTC* Papillary thyroid cancer, *SD* Standard deviation

^1^Calculated by Chi-squared test for categorical variables

^a^For controls, the age at diagnosis year of matched case

^b^For controls, the time between serum sample collection and age at diagnosis year of matched case

### Laboratory analysis

Serum concentrations of nine OCPs, including *p,p’*-DDT and its metabolites *p,p’*-DDE and *o,p’*-DDT, HCB, chlordane metabolites (oxychlordane and *trans*-nonachlor), lindane metabolites (γ-HCCH and β-HCCH), and mirex were measured in the earliest pre-diagnostic serum sample, collected on average 8.5 years prior to the PTC diagnosis (1994–2010) for cases. All chemical measurements were performed at the Persistent Organic Pollutants Laboratory at the Centers for Disease Control and Prevention (Atlanta, Georgia) using previously described procedures [33, 54, 72]. Briefly, serum sample processing included automatic fortification of sera using internal standards, as well as addition of formic acid and water for denaturation and sample dilution using Gilson 215 liquid handler (Gilson Inc.; Middleton, Wisconsin). Samples were then extracted by automated liquid-liquid extraction using the liquid handler. Removal of co-extracted lipids was performed on a silica/sulfuric acid column using the Rapid Trace equipment for automation (Biotage; Uppsala, Sweden). Final analytical determination of the target OCP analytes was performed by gas chromatography isotope dilution high resolution mass spectrometry employing a DFS instrument (ThermoFinnigan MAT; Bremen, Germany) [72]. Total triglycerides and total cholesterol were also measured in each serum sample using commercially available test kits (Roche Diagnostics Corp.; Indianapolis, Indiana) and a Hitachi 912 Chemistry Analyzer (Hitachi; Tokyo, Japan). These lipid measurements were used to calculate total serum lipid concentrations (mg/dL serum) using previously described methods [60]. Serum concentrations of all OCP analytes were reported as lipid-corrected concentrations (ng/g lipid weight) after subtracting the median concentration of contaminants measured in blank samples.

Laboratory personnel were blinded to the case-control status of study participants. Internal laboratory controls included method blanks (*n* = 3) and quality controls (*n* = 3) in every set of 24 study samples. The coefficient of variation for the OCPs measured in quality control samples ranged from 2.8% (trans-nonachlor) to 9.2% (γ-HCCH).

### Statistical analyses

We compared distributions of the demographic and military characteristics between cases and controls using two-sided Chi-squared tests. Due to very low detection frequencies for γ-HCCH (0.55%>limit of detection [LOD]) and *o,p’*-DDT (1.44%>LOD overall), we excluded these OCPs from all analyses. For most chemicals with at least 40% >LOD overall (*p,p’*-DDT, HCB, oxychlordane, *trans*-nonachlor), we categorized concentrations into three serum concentration tertiles ≥ LOD and the comparison category of < LOD. Because *p,p’*-DDE was detected in 99.9% of the samples, we categorized it into quartiles, with the lowest quartile serving as the reference group. Despite their relatively lower detection, we evaluated β-HCCH (20.53%>LOD overall) and mirex (14.85%>LOD overall) using categorical analyses because these congeners or isomers of these congeners were included in past studies [15, 41, 42]. We categorized the concentrations of β-HCCH and mirex as detected when the measured concentration was greater than or equal to the LOD or as non-detected where the concentration was less than the LOD.

To examine associations between the categories of individual OCPs and PTC risk, we used conditional logistic regression and estimated odds ratios (ORs) and 95% CIs. Each model was adjusted for the military branch of service (Army, Air Force, Navy, Marines or Coast Guard) and body mass index (BMI) categories (< 25 kg/m^2^, 25-29.9 kg/m^2^, ≥ 30 kg/m^2^, missing). To investigate dose-response relationships, we calculated *p*-values for tests for pseudo-linear trend by modeling median analyte concentration within each category of exposure as a continuous variable. We conducted stratified analyses by histological subtype (classical PTC, follicular variant PTC) and further within classical PTC cases and their matched controls by sex (male, female), birth year cohort (≤ 1970, > 1970), and race (non-Hispanic White, non-Hispanic Black). We calculated *p*-values from Wald’s test of OR homogeneity across case histological subtypes using case-only unconditional logistic regression models adjusted for age at PTC diagnosis, race/ethnicity, sex, BMI, and military branch. Stratified by sex, birth year, and race, we calculated p-interaction values for multiplicative interaction between categories of each chemical exposure and stratification variable. A sensitivity analysis was conducted by excluding diagnosed PTC cases within five years since serum sample collection and their matched controls, to account for the potential latent time period of PTC development.

### Comparison to NHANES

We drew comparisons to the National Health and Nutrition Examination Survey (NHANES),  a nationally representative survey and examination that assesses the health and nutritional status of the civilian U.S. population [10], to provide context to the levels reported in our study population. Included within the survey is a measurement of specific chemicals and their metabolites in both blood and urine, which can be used for environmental exposure assessment. Details of the methods are described elsewhere [10]. Surveys are conducted in two-year cycles; therefore, we selected the 2003–2004 cycle as it represented the approximate midpoint for the serum samples collected in our study (1994–2010). We restricted the NHANES population to ages 17–52 (*n* = 1016), representing the range of ages in our military population. The NHANES data are publicly available: https://wwwn.cdc.gov/nchs/nhanes/search/datapage.aspx?Component=Laboratory&Cycle=2003-2004. We present the %>LOD, median, IQR, maximum, and LOD values for the military cases, military controls, and NHANES 2003–2004 population for visual comparison between the distributions of the measured OCPs. 

## Results

The characteristics of cases and controls are presented in Table 1. The distribution of the military service branch significantly differed by the case-control status. Cases were more likely to have served in the Army or the Air Force at the time of diagnosis and less likely to have served in the Navy or the Marines/Coast Guard (*p* < 0.0001). A higher proportion of cases than controls had BMI of at least 25 kg/m^2^ (22.1.0% vs. 18.7%); however, BMI information was missing for 43.3% of the cases and 42.9% of the controls and the difference in the BMI distribution did not statistically differ by case-control status (*p* = 0.17). The distributions of age, sex, and race/ethnicity did not differ between cases and controls due to matching. The mean age of cases at time of diagnosis was 35.1 years (standard deviation [SD] = 8.1) and mean age at serum sample draw was 27.3 years (SD = 7.4) for cases and 27.2 years (SD = 7.3) for controls. Nearly half of cases (46%) were female, 63.1% identified as non-Hispanic white, 17.6% as non-Hispanic Black, 9.2% as Hispanic, and the rest were people of other or unknown race/ethnicity. On average, serum samples were collected 8.4 years (SD = 2.7) before the PTC diagnosis. Most PTC cases had tumor size of > 10 mm (62.4%) and were classified as classical PTC histological subtype (80.9%).

Lipid-corrected serum concentrations of all the OCPs measured in this study are presented in Table 2. The detection frequencies and median OCP concentrations were similar between cases and controls. For the sake of descriptive comparison, we also present the NHANES OCP concentrations for 2003–2004. Detection frequency for all congeners, with the exception of *p,p’*-DDE, was higher in the NHANES population than the military population. Median and 75th percentile HCB concentrations were greater among NHANES participants (14.00 and 17.4 ng/g lipid, respectively) than among our study population cases (1.61 and 7.52 ng/g lipid, respectively) and controls (< LOD and 7.2 ng/g lipid, respectively). Similarly, median concentrations of *p,p’*-DDE (165.00, 133.30, and 129.70 ng/g lipid, respectively), oxychlordane (7.7 ng/g lipid, 5.3 ng/g lipid, and < LOD, respectively), and trans-nonachlor (11.50, 8.70, and 7.40 ng/g lipid, respectively) concentrations were also higher among NHANES participants than the cases and controls in our military study population.

**Table 2:** Lipid-corrected serum concentrations and limit of detection (LOD) of organochlorine pesticides (ng/g lipid) in study population (sample collection 2000–2013)^a^ and NHANES (2003–2004)^b^

OCP	Overall		Cases (*N* = 742), age 17–52 years				Controls (*N* = 742), age 17–52 years				NHANES 2003–2004 (*N* = 1016), age 17–52 years^d^				
	% >LOD	LOD	% >LOD	Median	IQR	Max	% >LOD	Median	IQR	Max	% >LOD	Median	IQR	Max	LOD
β-HCCH	20.5%	4.9	22.3%	<LOD	<LOD - <LOD	371.4	18.8%	<LOD	<LOD - <LOD	153.8	60.7%	4.3	2.47–8.10	456	2.3
γ-HCCH^c^	0.6%	5.0	0.6%	<LOD	<LOD - <LOD	5277	0.6%	<LOD	<LOD - <LOD	494.4	0.9%	<LOD	<LOD - <LOD	73.6	2.1
HCB	48.5%	4.9	50.0%	1.61	<LOD − 7.52	150.7	47.1%	<LOD	<LOD-7.20	40.81	100%	14.0	10.80–17.40	174	2.2
Mirex	14.9%	4.9	14.6%	<LOD	<LOD - <LOD	125.4	15.1%	<LOD	<LOD - <LOD	348.2	26.3%	<LOD	<LOD - <LOD	166	2.2
*o,p’*-DDT^c^	1.4%	4.9	1.6%	<LOD	<LOD - <LOD	26.7	1.2%	<LOD	<LOD - <LOD	82.9	5.2%	<LOD	<LOD - <LOD	88.8	2.1
*p,p’*-DDT	40.1%	4.9	41.2%	<LOD	<LOD-7.1	126.8	38.9%	<LOD	<LOD-6.50	87.1	69.9%	3.96	2.69–5.90	676	2.3
*p,p’*-DDE	99.9%	4.9	100%	133.3	82.1-230.7	1882	99.9%	129.7	78.6-233.5	2049	99.7%	165	97.30–318.00	15,100	2.9
Oxychlordane	51.2%	4.9	54.1%	5.3	<LOD-10.3	104.8	48.4%	<LOD	<LOD-9.90	40.9	73.2%	7.7	4.10–12.60	61.4	2.5
*trans*-nonachlor	66.9%	4.9	68.7%	8.7	<LOD-16.4	304.3	65.1%	7.4	<LOD-14.50	89.2	89.4%	11.5	6.60–19.80	121	2.5

*Abbreviations:* *DDE* Dichlorodiphenyldichloroethylene, *DDT* Dichlorodiphenyltrichloroethane, *IQR* Interquartile range, *LOD* Limit of detection, *NHANES* National Health and Nutrition Examination Survey

^a^Study population median (IQR) age at sampling: 35 (29–41) years

^b^NHANES median (IQR) age at sampling (age restricted to 17–52 years): 31 (21–41) years

^c^ Excluded from any regression analyses

^d^NHANES data adjusted for sampling weights

Table 3 presents the ORs and 95% CIs for PTC risk with exposure to the various OCPs included in our analyses; we first present results for all PTC cases and their matched controls and then present results stratified by histological subtype: classical PTC and follicular PTC. In analyses including all PTC cases, we did not find evidence of a significant association between any of the OCPs and PTC risk. However, after stratifying by histological subtype, there was a significantly increased risk for classical PTC for the third tertile > LOD of HCB compared to the < LOD category (OR = 1.61, 95%CI: 1.09, 2.38; *p*-trend = 0.05) and a significantly decreased risk for follicular PTC in the highest two categories of HCB (OR_highest_ = 0.38, 95%CI: 0.16, 0.91; *p*-trend = 0.04), indicating a difference in effect by histological subtype, though the Wald’s test of OR homogeneity was not statistically significant (*p*-Wald = 0.20). For both oxychlordane and *trans*-nonachlor there appeared to be patterns of elevated ORs with increasing serum concentration categories in the classical PTC stratum, though estimates were not statistically significant. There also appeared to be patterns of decreased ORs with increasing serum concentration categories for each OCP for follicular PTC, but estimates were not statistically significant, with the exception of HCB (*p*-trend = 0.04). Wald’s tests of OR homogeneity across histological subtype were not statistically significant, except for *p,p’*-DDT (*p*-Wald = 0.03).

**Table 3:** Associations^a^ between lipid-corrected serum concentrations of organochlorine pesticides and risk of papillary thyroid cancer, overall and stratified by histological subtype

	Overall				Stratified by histological subtype								
	742 case/control pairs				Classical PTC 600 case/control pairs				Follicular PTC 142 case/control pairs				
OCP levels (ng/g lipid)	N cases	N controls	OR	95% CI	N cases	N controls	OR	95% CI	N cases	N controls	OR	95% CI	*p*-Wald^c^
*p,p’*-DDE													
<LOD-78.56	174	185	1.00	Ref.	137	152	1.00	Ref.	37	33	1.00	Referent	
78.57-129.69	179	186	0.98	(0.72–1.34)	142	150	1.04	(0.73–1.49)	37	36	0.84	(0.44–1.61)	
129.70-233.49	207	185	1.11	(0.80–1.53)	172	148	1.24	(0.86–1.78)	35	37	0.74	(0.36–1.55)	
233.50-2,049.00	182	186	0.93	(0.64–1.34)	149	150	1.02	(0.67–1.56)	35	36	0.62	(0.28–1.38)	
p-trend				0.87				0.96				0.68	0.58
*p,p’*-DDT													
<LOD	429	446	1.00	Ref.	336	366	1.00	Ref.	93	80	1.00	Referent	
≥LOD-6.30	84	94	0.91	(0.65–1.27)	69	76	0.98	(0.68–1.42)	15	18	0.58	(0.25–1.34)	
6.31–10.35	115	95	1.17	(0.82–1.67)	104	80	1.37	(0.93–2.01)	11	15	0.44	(0.15–1.33)	
10.36–126.80	102	95	1.03	(0.72–1.47)	84	70	1.31	(0.87–1.98)	18	25	0.48	(0.21–1.06)	
p-trend				0.94				0.94				0.63	**0.03**
HCB													
<LOD	361	379	1.00	Ref.	283	315	1.00	Ref.	78	64	1.00	Referent	
≥LOD-6.36	124	112	1.13	(0.82–1.54)	105	95	1.22	(0.86–1.71)	19	17	0.77	(0.33–1.80)	
6.37–8.64	99	112	0.87	(0.62–1.22)	80	85	1.03	(0.71–1.51)	19	27	**0.38**	**(0.17–0.89)**	
8.65–150.70	138	113	1.23	(0.87–1.75)	119	86	**1.61**	**(1.09–2.38)**^**b**^	19	27	**0.38**	**(0.16–0.91)**	
p-trend				0.41				**0.05**				**0.04**	0.20
Oxychlordane													
<LOD	340	381	1.00	Ref.	269	310	1.00	Ref.	71	71	1.00	Referent	
≥LOD-7.95	127	118	1.17	(0.85–1.61)	103	97	1.23	(0.87–1.75)	24	21	0.89	(0.40–1.99)	
7.96–12.65	138	119	1.25	(0.90–1.74)	115	97	1.35	(0.94–1.95)	23	22	0.77	(0.33–1.77)	
12.66–104.80	135	120	1.21	(0.85–1.72)	111	92	1.36	(0.92–2.01)	24	28	0.60	(0.25–1.47)	
p-trend				0.24				0.12				0.65	0.75
*trans*-Nonachlor													
<LOD	231	259	1.00	Ref.	183	209	1.00	Ref.	48	50	1.00	Referent	
≥LOD-8.92	150	160	1.04	(0.77–1.40)	115	132	0.98	(0.70–1.37)	35	28	1.11	(0.54–2.27)	
8.93–15.84	164	161	1.18	(0.86–1.63)	141	131	1.29	(0.91–1.83)	23	30	0.65	(0.27–1.56)	
15.85–304.30	194	162	1.36	(0.96–1.92)	159	128	1.45	(0.99–2.13)	35	34	0.78	(0.32–1.94)	
p-trend				0.12				0.085				> 0.99	0.16
β-HCCH													
<LOD	566	595	1.00	Ref.	448	477	1.00	Ref.	118	118	1.00	Referent	
≥LOD (2.77–153.80)	162	138	1.12	(0.84–1.50)	142	115	1.21	(0.88–1.66)	20	23	0.71	(0.32–1.57)	0.16
Mirex													
<LOD	624	620	1.00	Ref.	505	502	1.00	Ref.	119	118	1.00	Referent	
≥LOD (2.88–348.20)	107	110	0.97	(0.70–1.34)	88	90	0.96	(0.67–1.38)	19	20	1.07	(0.50–2.30)	0.97

Bolding denotes statistical significance (*p*<0.05)

*Abbreviations: CI* Confidence interval, *DDE* Dichlorodiphenyldichloroethylene, *DDT* Dichlorodiphenyltrichloroethane, *HCB* Hexachlorobenzene, *HCCH* Hexachlorocyclohexane, *LOD* Limit of detection, *OR* Odds ratio, *PTC* Papillary thyroid cancer

^a^All models adjusted for BMI (< 25 kg/m2, 25–29.9 kg/m2, ≥ 30 kg/m^2^, missing) and military branch (Army, Air Force, Navy, Marines/Coast Guard)

^b^When top (3rd) tertile was split at the median into lower median (3rd tertile-LM) and upper median (3rd tertile-UM): OR_3rd tertile-LM_ = 1.43; 95%CI, 0.87–2.36 and OR_3rd tertile-UM_ = 1.76; 95%CI, 1.10–2.82; *p*-trend = 0.04

^c^*P*-value from a Wald test of OR homogeneity across case histological subtype from case-only unconditional logistic regression models from case-only analyses adjusting for age at PTC diagnosis, race/ethnicity, sex, BMI, and military branch

Results from our stratified analyses by sex for classical PTC are presented in Table 4. Counts of follicular PTC were too small to carry out further stratified analyses for that histological subtype. There was a significantly increased risk for classical PTC in the third concentration tertile > LOD of HCB among females only (OR = 2.23, 95%CI: 1.23, 4.06; *p*-trend = 0.02). Additionally, there was significantly increased risk for classical PTC among females with β-HCCH concentrations greater than or equal to the LOD (OR = 1.76, 95% CI: 1.07, 2.89). The second concentration tertile of trans-nonachlor > LOD was associated with a significant increased risk of classical PTC among males (OR = 1.66, 95% CI: 1.02, 2.70; p-trend = 0.07), but otherwise ORs were relatively null for males, there were not consistent patterns of increased or decreased risk, and there was no evidence of significant effect modification by sex based on *p*-values for interaction.

**Table 4:** Associations between lipid-corrected serum concentrations of organochlorine pesticides and risk of classical papillary thyroid cancer, stratified by sex

OCP levels (ng/g lipid)	Males: 325 case/control pairs				Females: 275 case/control pairs				*p*-interaction
	N _cases_	N _controls_	OR	95% CI	N _cases_	N _controls_	OR	95% CI	
*p,p’*-DDE									
<LOD-78.56	66	73	1.00	Ref.	71	79	1.00	Ref.	
78.57-129.69	82	80	1.01	(0.62–1.67)	60	70	1.01	(0.59–1.73)	
129.70-233.49	97	90	1.03	(0.63–1.71)	75	58	1.52	(0.88–2.63)	
233.50-2,049.00	80	82	0.84	(0.47–1.49)	69	68	1.27	(0.67–2.39)	
*p*-trend				0.55				0.59	0.76
*p,p’*-DDT									
<LOD	178	192	1.00	Ref.	158	174	1.00	Ref.	
≥LOD-6.30	47	47	0.96	(0.60–1.53)	22	29	0.91	(0.49–1.71)	
6.31–10.35	51	43	1.05	(0.61–1.80)	53	37	1.70	(0.97–2.98)	
10.36–126.80	47	40	1.18	(0.68–2.05)	37	30	1.50	(0.80–2.84)	
*p*-trend				0.61				0.51	0.78
HCB									
<LOD	148	158	1.00	Ref.	135	157	1.00	Ref.	
≥LOD-6.36	66	64	1.01	(0.64–1.60)	39	31	1.57	(0.92–2.68)	
6.37–8.64	47	43	1.05	(0.63–1.74)	33	42	1.01	(0.56–1.83)	
8.65–150.70	59	49	1.22	(0.72–2.08)	60	37	**2.23**	**(1.23–4.06)**	
*p*-trend				0.56				**0.02**	0.30
Oxychlordane									
<LOD	130	158	1.00	Ref.	139	152	1.00	Ref.	
≥LOD-7.95	63	60	1.21	(0.76–1.91)	40	37	1.22	(0.71–2.10)	
7.96–12.65	67	51	1.43	(0.88–2.32)	48	46	1.24	(0.70–2.18)	
12.66–104.80	64	55	1.21	(0.71–2.04)	47	37	1.47	(0.81–2.68)	
*p*-trend				0.28				0.27	0.86
*trans*-Nonachlor									
<LOD	81	105	1.00	Ref.	102	104	1.00	Ref.	
≥LOD-8.92	61	76	0.92	(0.58–1.47)	54	56	0.99	(0.61–1.63)	
8.93–15.84	85	66	**1.66**	**(1.02–2.70)**	56	65	0.92	(0.56–1.57)	
15.85–304.30	98	78	1.56	(0.93–2.60)	61	50	1.22	(0.70–2.29)	
*p*-trend				**0.07**				0.56	0.26
β-HCCH									
<LOD	246	250	1.00	Ref.	202	227	1.00	Ref.	
≥LOD (2.77–153.80)	74	68	0.88	(0.57–1.35)	68	47	**1.76**	**(1.07–2.89)**	0.10
Mirex									
<LOD	266	265	1.00	Ref.	239	237	1.00	Ref.	
≥LOD (2.88–348.20)	57	57	1.03	(0.65–1.63)	31	33	0.90	(0.50–1.62)	0.73

All models adjusted for BMI (< 25 kg/m2, 25–29.9 kg/m2, ≥ 30 kg/m^2^, missing) and military branch (Army, Air Force, Navy, Marines/Coast Guard)

Bolding denotes statistical significance

*Abbreviations: CI* Confidence interval, *DDE* Dichlorodiphenyldichloroethylene, *DDT* Dichlorodiphenyltrichloroethane, *HCB* Hexachlorobenzene, *HCCH* Hexachlorocyclohexane, *LOD* Limit of detection, *OR* Odds ratio

Classical PTC cases and controls stratified by birth year (≤ 1970, > 1970) and race (non-Hispanic White, non-Hispanic Black) are presented in Supplemental Table 1. There were some statistically significant findings and increased or decreased ORs, but there were no clear trends or statistically significant findings of interaction. After restricting the study population to cases diagnosed at least five years after serum sample collection and their matched controls, the ORs for all cancers, for classical PTC, and for classical PTC among females, were somewhat attenuated but similar to those presented in Tables 3 and 4 (Supplemental Table 2).

## Discussion

In this study carried out in a relatively young military population, we did not find associations between serum *p,p’*-DDE, *p,p’*-DDT, HCB, oxychlordane, trans-nonachlor, β-HCCH, and mirex concentrations and overall PTC risk. However, when stratified by histological subtype, we found a suggestion of increased risk for classical PTC and decreased risk for follicular PTC with elevated serum concentrations of HCB. People in the highest concentration tertile (third tertile > LOD) were 60% more likely to develop classical PTC than people in the reference group (< LOD). Risk for follicular PTC was decreased among people in the highest two concentration tertiles > LOD of HCB compared to < LOD, but as there were only 142 follicular PTC pairs, these results should be interpreted with caution, and we were not able to further evaluate this finding via additional stratified analyses. Further stratified by sex, classical PTC risk was higher in females in the highest HCB concentration category (third tertile > LOD), compared to females in the lowest concentration category (< LOD) and findings were null among men; however, tests for effect modification by sex were not statistically significant. Females with β-HCCH concentrations greater than or equal to the LOD compared to the reference group (< LOD) were 76% more likely to develop classical PTC, with findings null among males; however, these results should be interpreted with caution given the low detection frequency of this isomer. We did not find evidence of increased risk for classical or follicular PTC with increasing serum concentrations of *p,p’*-DDE, *p,p’*-DDT, oxychlordane, *trans*-nonachlor, or mirex. Additional stratification by birth cohort indicated the possibility for a stronger risk among those born more recently and thus with earlier life exposures to HCB and oxychlordane; however, tests for effect modification were not statistically significant.

Although the potential health effects of exposure to HCB and other OCPs are not entirely understood, HCB has been shown to reduce viability and inhibit cell growth in thyroid epithelial cells [40] and has been associated with an increase in thyroid tumor incidence in hamsters [8]. Hypermethylation of the MGMT gene, which has been associated with several human cancers, following OCP exposure has been reported; therefore, PTC occurrence could be related to epigenetic alterations [69].

Although the literature evaluating the relationship between changes in certain thyroid hormone levels and thyroid cancer risk is mixed, HCB, β-HCCH, and trans-nonachlor could influence thyroid cancer risk through potential changes in thyroid hormone function. Some studies have observed an association between high levels of serum free thyroxine (T4) with incident thyroid cancer [24, 36], though previous work in our study population did not support these findings [25]. HCB and β-HCCH have been reported to be associated with reduced total serum T4 [39, 68] levels in humans and animals, with other research noting differences by sex [19], and HCB has been reported to be competitive with T4 binding to a major plasma transporting protein in rats [81]. However, positive relationships between free T4 serum levels and HCB have been observed in females [19] and female sex has been associated with increased HCB concentrations compared to males [3, 14, 59]. Additionally, inverse relationships between serum total triiodothyronine (T3) levels and HCB have been noted in humans [48]. Previously, in this study population we found an inverse association between serum T3 levels above the normal range and risk of PTC among males (OR: 0.59; 95%CI: 0.36, 0.98), while the risk of PTC increased with increasing serum concentrations of T3 among females (overall *p*-trend = 0.019) [25]. Similarly, inverse relationships with blood levels of total T3 and β-HCCH have been noted in humans [2]. Further study is needed to elucidate the mechanisms behind thyroid cancer etiology and sex-specific differences in the relationship between OCP exposure and thyroid cancer.

The results from our study support some findings from prior studies and contradict those from other studies. A nested case-control study based in Norway observed elevated odds of thyroid cancer associated with exposure to a sum of chlordane metabolites (i.e., oxychlordane and trans-nonachlor in serum) and a stronger risk among participants born between 1943–1957 but not earlier birth cohorts (1923–1932, 1933–1942) [42]. DDT exposure was also inversely associated with thyroid cancer (OR = 0.78, 95% CI: 0.62, 0.97 per 1 ng/g increase in DDT). The Norwegian study did not observe a positive association between HCB or *β*-HCCH, even when stratified by sex. Like our study, the Norwegian study used pre-diagnostic blood samples to assess OCP exposure; however, the study population was smaller and older compared to our population, and median serum concentrations of *p,p’*-DDE, trans-nonachlor, and HCB were higher. A study evaluating self-reported pesticide use and incident thyroid cancer among male pesticide applicators in the Agricultural Health Study (AHS) observed use of lindane, an HCCH isomer, to be significantly associated with increased risk of thyroid cancer (hazard ratio [HR] = 1.74, 95%CI: 1.06, 2.84) when comparing ever users to never users, but when restricted to PTC specifically, findings were not statistically significant (HR = 1.64, 95%CI: 0.93, 2.88) [41]. Our study observed a relationship between β-HCCH and PTC only among female service members, though we evaluated a different HCCH isomer from the AHS. However, lindane used as insecticide may contain alpha and beta isomers in addition to γ-HCCH [51]. These conflicting findings may relate to differences in study populations or the use of questionnaires to estimate OCP exposure in the AHS. In a study of AHS female spouses of insecticide applicators, Louis et al. [45] did not observe any significant associations between self-reported OCP use and thyroid cancer when comparing ever use to never use [45]. Another study conducted by Grimalt et al. [21] detected unusually high concentrations of HCB in the air and sera of volunteers in the village of Flix, Spain, and observed a higher incidence of thyroid cancer in males [age-standardized incidence ratio (SIR) = 6.7, 95%CI: 1.6, 28.0], but not females (SIR = 1.0, 95%CI: 0.14, 7.4) [21]; however, this report included a small sample size and 95% CIs were wide. Cancer incidence from 1980 to 1989 was obtained from the cancer registry covering the village, whereas our study evaluated thyroid cancer diagnoses from 2000 to 2013. Additionally, the community in the Spanish study was highly exposed to HCB through air pollution and for many residents, occupationally. The contrasting findings by sex between the Spanish study and our study could be partially attributed to differences in occupational histories, with Grimalt et al. [21] noting most male questionnaire respondents with cancer had been employed at the factory and no female respondents had been. Finally, a case-control study evaluating the relationship between serum concentrations of similar OCP congeners and PTC among Connecticut (USA) females diagnosed between 2010 to 2011 did not observe positive associations with any OCPs in single or multi-pollutant models [15]. Like our study, the Connecticut study was based in the USA; however, our study population was younger, larger, and more racially and ethnically diverse, and our study had pre-diagnostic samples rather than post-diagnostic samples. Studies evaluating cancer risk and employing post-diagnostic sampling may be particularly susceptible to reverse causation, confounding, selection bias, and information bias [13]. Studies with post-diagnostic samples may also be particularly susceptible to exposure misclassification, as there may be changes in exposure pre- versus post-diagnosis due to disease progression impacting exposure [13]. Additionally, median HCB, oxychlordane, and trans-nonachlor serum concentrations were higher in the Connecticut study, while median *p,p’*-DDE concentrations were higher in our study population. Inconsistent epidemiologic evidence across studies evaluating associations between OCP exposure and thyroid cancer risk could be related to small study sizes, different age distributions, post-diagnostic samples of OCPs, or the use of proxies to estimate exposure (i.e., ascertaining pesticide use through questionnaires).

To our knowledge, this is the first study to observe differences in PTC risk associated with HCB exposure by classical and follicular variant. This may be partially explained by the small counts of participants diagnosed with the follicular variant. There have been differences in the gene mutations noted between classical and follicular variants [52], suggesting differing etiologies. For example, BRAF V600E and RAS mutations are the main genetic drivers in thyroid cancers, followed by fusions involving RET and other receptor tyrosine kinases. BRAFV600E mutation is more common in classical PTC while RAS mutations occur more in follicular variant PTC [62]. Our findings warrant further investigation in studies with a greater number of follicular variant PTC cases.

Although the latent period of thyroid cancer, and PTC specifically, is largely unknown, it has been suggested to be approximately 5 to 10 years after radiation exposure [31]. Therefore, we conducted a sensitivity analysis including only cases with serum samples collected 5 or more years prior to PTC diagnosis and their matched controls. Overall, findings were similar, though in some cases ORs were slightly attenuated, suggesting that the latency of PTC did not meaningfully impact our results. OCPs have relatively long half-lives and are persistent in the environment, so it may be argued that concentrations measured in more recent serum samples may provide an estimate of body burden from earlier time periods.

The median serum concentrations of HCB, *p,p’*-DDE, oxychlordane, and *trans*-nonachlor in our study were lower than those in a U.S. general population from a similar age range (17–52 years) and time period (2003–2004) that was approximately the midpoint of our sample collection period (1994–2010). In particular, concentrations of HCB appear to be much lower in our study population compared to the NHANES population. There are a few potential explanations for these differences in findings. First, despite our attempt to include the same age ranges between the two populations, there was a larger distribution of older people in the NHANES sample (approximately 30% between the ages of 40 and 52) than in the military study population (approximately 7% between the ages of 40 and 52), and older age has been associated with higher concentrations of OCPs [5, 32, 35, 47, 70, 75]. Second, we would expect higher concentrations of serum OCPs in the NHANES population compared to the military population because our military study population was mostly non-Hispanic White (63.1% cases, 62.9% controls) and less than 10% of the study population was Hispanic of any race, while the NHANES study population was approximately 45% non-Hispanic White, 22% Mexican-American, and 3.64% other Hispanic. Concentrations of OCPs have been reported to be higher in Mexican American persons than both non-Hispanic White and non-Hispanic Black persons [43]. Additionally, our military study population was 54% male and 46% female, while the NHANES population included fewer males than females (approximately 48% male and 52% female). These distributions are similar; however, some research has shown higher concentrations of OCPs in females compared to males [4, 35, 61]. The years samples were drawn also differed between the NHANES (2003–2004) and the military (1995–2010) populations and past studies have suggested declines in OCP body burdens over time [43]. However, even when we restricted the military study population to participants with serum samples from 2003–2004, median OCP concentrations were higher in the 2003–2004 NHANES; however, this is based on a small number of military participants (*n* = 120).

Despite the lower concentrations from our military population, compared with NHANES, environmental studies of fish, wildlife, and soil surrounding military sites and FUDS, suggest military-related OCP contamination [7, 20, 50, 53, 64, 66, 71, 88]. Although evidence suggests the potential for increased exposure around DoD sites, findings from our study do not provide evidence of elevated OCP exposure among U.S. military service members, compared to the general U.S. population, though this could vary by geographic location and specific job duties.

The present study has several strengths. This study had a relatively large sample size, which offered sufficient statistical power to stratify by histological subtype and demographics, such as sex, race, and birth cohort. Given that females have an approximately three-times higher rate of thyroid cancer than males [55], examining associations by sex is important. Second, the study population was composed of U.S. active-duty military personnel, a younger population, well representing age groups most affected, as the incidence of PTC is highest among patients aged approximately 30–50 years [12]. Additionally, the universal military healthcare system minimizes the potential selection bias stemming from differences in healthcare access. Third, the concentrations of OCPs were assessed prospectively in the DoDSR cohort through pre-diagnostic samples, which enabled us to evaluate a temporal relationship between exposure and outcome. Lastly, a major strength of our study was the use of pre-diagnostic serum samples for measurements of OCP exposures, which minimized the potential for reverse causality. Given that the latency period of thyroid cancer has been observed to be approximately 5–10 years [31], the average 9 years between serum samples of OCPs measurement and the diagnosis of PTC cases was appropriate.

There were several limitations to this work. First, there was a lack of information on potential confounders, including ionizing radiation exposure, thyroid disease history, and family history of thyroid cancer. Additionally, a high percentage of participants were missing BMI data; therefore, we may not have sufficiently adjusted for obesity, an established PTC risk factor [37, 46, 56, 87]. However, prior work has suggested lower prevalence of obesity and larger lean body mass among military personal compared to the general U.S. population [73], so residual confounding of BMI would likely be minimized. Second, we could not stratify analyses for the follicular variant of PTC as the overall number of participants with follicular variant PTC was small. Results we present here for the follicular variant should be interpreted with caution, due to the low sample size of this group. Additionally, findings related to β-HCCH and mirex and classical PTC risk should be interpreted with caution due to their relatively low detection frequencies, which may relate to the small serum volume of 0.5 mL available for our study, which is the maximum size of aliquot permitted by the AFHSD to be utilized for a given sample from the DoDSR [57, 58]. Finally, OCP concentrations are based on a single serum measurement; however, given the long biological half-lives of most OCPs (i.e., order of years) [6], use of a single serum sample is considered to adequately represent OCP exposure over time [49]. The single measurement used for serum OCP concentrations suggested that OCP exposures are lower in this study population than the US general population, represented by NHANES, which may contribute to our generally null findings.

In conclusion, in this large, nested case-control study we observed a 60% increased risk of classical PTC in the highest exposure group of HCB (8.65–150.70 ng/g-lipid) compared to the lowest exposure group (< LOD), with a statistically significant dose-response. Sex appeared to modify this risk, with a greater than two-fold increased risk among females and a close to null effect for men, though the effect did not reach statistical significance. Additionally, β-HCCH concentration ≥ LOD was associated with a 76% increased risk of classical PTC compared to the reference group < LOD. The fact that HCB concentrations in our study were lower than the general U.S. population indicates the need for further studies to elucidate the contribution of HCB to PTC risk over the range of exposures to the U.S. population. Collectively, these findings are provocative and should be further studied to better elucidate the relationship between OCP exposures and thyroid cancer risk in humans, to better understand disparities between males and females, and to gain a better understanding of the risks of early life exposures.

### Supplementary Information

**Supplementary Material 1.**

## Data Availability

Data are available upon reasonable request. Since these data are from military data resources, the process for obtaining assurances for public use requires authorisations by the Department of Defense. The senior author can provide additional information on the process upon request.

## Abbreviations

ACTUR: Automated Central Tumor Registry
AHS: Agricultural Health Study
BMI: Body mass index
CI: Confidence interval
DDE: Dichlorodiphenylchloroethylene
DDT: Dichlorodiphenyltrichloroethane
DMSS: Defense Medical Surveillance System
DoD: Department of Defense
DoDSR: Department of Defense Serum Repository
EDC: Endocrine disrupting chemical
EPA: Environmental Protection Agency
FUDS: Formerly used defense sites
HCB: Hexachlorobenzene
HCCH: Hexachlorocyclohexane
HR: Hazard ratio
ICD-O-3: International Classification of Diseases for Oncology
LOD: Limit of detection
NHANES: National Health and Nutrition Examination Survey
OCP: Organochlorine pesticide
OR: Odds ratio
PTC: Papillary thyroid cancer
SD: Standard deviation
SIR: Standardized incidence ratio
T3: Triiodothyronine
T4: Thyroxine
