# Supplementary material for: Organochlorine pesticides and risk of papillary thyroid cancer in U.S. military personnel: a nested case-control study
Source: Environ Health. 2024 Mar 19;23:28. doi: 10.1186/s12940-024-01068-0 (PMC10949709; doi:10.1186/s12940-024-01068-0)
Supplement: Supplementary file 1 — Supplementary Material 1. [file 12940_2024_1068_MOESM1_ESM.docx]

Supplemental Tables

| Supplemental Table 1. Associations between lipid-corrected serum concentrations of organochlorine pesticides and risk of classical papillary thyroid cancer, stratified by birth year and by race | | | | | | | | | | | | | | | | | | |
| --- | --- | --- | --- | --- | --- | --- | --- | --- | --- | --- | --- | --- | --- | --- | --- | --- | --- | --- |
|  | Stratified by birth year | | | | | | | |  | Stratified by race | | | | | | | |  |
|  | ≤1970 (253 cases/251 controls) | | | | >1970 (347 cases/349 controls) | | | |  | NH White (386 cases/385 controls) | | | | NH Black (100 cases/101 controls) | | | |  |
| OCP levels (ng/g lipid) | N_cases_ | N_controls_ | OR | 95% CI | N_cases_ | N_controls_ | OR | 95% CI | p-int | N_cases_ | N_controls_ | OR | 95% CI | N_cases_ | N_controls_ | OR | 95% CI | p-int |
| *p,p'*-DDE |  |  |  |  |  |  |  |  |  |  |  |  |  |  |  |  |  |  |
| <LOD-78.56 | 20 | 19 | 1.00 | Ref. | 117 | 133 | 1.00 | Ref. |  | 96 | 112 | 1.00 | Ref. | 22 | 16 | 1.00 | Ref. |  |
| 78.57-129.69 | 48 | 47 | 0.97 | (0.44-2.13) | 94 | 103 | 1.07 | (0.71-1.63) |  | 103 | 111 | 1.06 | (0.69-1.62) | 20 | 22 | 0.64 | (0.23-1.79) |  |
| 129.70-233.49 | 79 | 79 | 0.90 | (0.42-1.91) | 93 | 69 | 1.51 | (0.97-2.35) |  | 119 | 100 | 1.42 | (0.91-2.22) | 27 | 22 | 0.74 | (0.27-2.07) |  |
| 233.50-2,049.00 | 106 | 106 | 0.79 | (0.36-1.74) | 43 | 44 | 1.18 | (0.65-2.14) |  | 68 | 62 | 1.27 | (0.74-2.18) | 31 | 41 | **0.26** | **(0.08-0.92)** |  |
| p-trend |  |  |  | 0.50 |  |  |  | 0.55 | 0.54 |  |  |  | 0.36 |  |  |  | **0.09** | 0.17 |
|  |  |  |  |  |  |  |  |  |  |  |  |  |  |  |  |  |  |  |
| *p,p'*-DDT |  |  |  |  |  |  |  |  |  |  |  |  |  |  |  |  |  |  |
| <LOD | 102 | 109 | 1.00 | Ref. | 234 | 257 | 1.00 | Ref. |  | 230 | 250 | 1.00 | Ref. | 48 | 46 | 1.00 | Ref. |  |
| ≥LOD-6.30 | 43 | 42 | 0.97 | (0.58-1.62) | 26 | 34 | 0.92 | (0.53-1.62) |  | 52 | 57 | 0.97 | (0.68-1.48) | 6 | 11 | 0.53 | (0.14-1.97) |  |
| 6.31-10.35 | 47 | 54 | 0.76 | (0.42-1.38) | 57 | 26 | **2.27** | **(1.32-3.91)** |  | 60 | 41 | 1.47 | (0.89-2.41) | 25 | 27 | 0.74 | (0.31-1.78) |  |
| 10.36-87.10 | 61 | 44 | 1.19 | (0.67-2.11) | 23 | 26 | 1.13 | (0.59-2.17) |  | 39 | 36 | 1.15 | (0.66-2.02) | 19 | 14 | 0.94 | (0.32-2.75) |  |
| p-trend |  |  |  | 0.48 |  |  |  | 0.43 | **0.03** |  |  |  | 0.37 |  |  |  | **0.09** | 0.52 |
|  |  |  |  |  |  |  |  |  |  |  |  |  |  |  |  |  |  |  |
| HCB |  |  |  |  |  |  |  |  |  |  |  |  |  |  |  |  |  |  |
| <LOD | 84 | 91 | 1.00 | Ref. | 199 | 224 | 1.00 | Ref. |  | 174 | 189 | 1.00 | Ref. | 55 | 63 | 1.00 | Ref. |  |
| ≥LOD-6.36 | 36 | 47 | 0.81 | (0.45-1.44) | 69 | 48 | **1.77** | **(1.13-2.78)** |  | 66 | 71 | 0.92 | (0.60-1.41) | 16 | 7 | **3.59** | **(1.18-10.93)** |  |
| 6.37-8.64 | 45 | 43 | 1.09 | (0.62-1.91) | 35 | 42 | 0.93 | (0.54-1.58) |  | 58 | 62 | 0.93 | (0.59-1.47) | 12 | 12 | 0.94 | (0.33-2.69) |  |
| 8.65-40.81 | 86 | 65 | 1.40 | (0.83-2.36) | 35 | 21 | 1.87 | (0.97-3.60) |  | 77 | 54 | 1.53 | (0.94-2.49) | 15 | 15 | 1.07 | (0.33-3.42) |  |
| p-trend |  |  |  | 0.27 |  |  |  | 0.11 | 0.11 |  |  |  | 0.22 |  |  |  | 0.47 | 0.10 |
|  |  |  |  |  |  |  |  |  |  |  |  |  |  |  |  |  |  |  |
| Oxychlordane |  |  |  |  |  |  |  |  |  |  |  |  |  |  |  |  |  |  |
| <LOD | 67 | 74 | 1.00 | Ref. | 202 | 236 | 1.00 | Ref. |  | 164 | 199 | 1.00 | Ref. | 46 | 44 | 1.00 | Ref. |  |
| ≥LOD-7.95 | 39 | 47 | 0.86 | (0.49-1.53) | 64 | 50 | **1.70** | **(1.06-2.72)** |  | 67 | 69 | 1.19 | (0.76-1.85) | 18 | 16 | 1.03 | (0.46-2.32) |  |
| 7.96-12.65 | 72 | 56 | 1.18 | (0.70-2.00) | 43 | 41 | 1.50 | (0.86-2.60) |  | 82 | 61 | **1.61** | **(1.03-2.54)** | 13 | 22 | 0.53 | (0.19-1.47) |  |
| 12.66-40.90 | 75 | 71 | 1.02 | (0.60-1.73) | 36 | 21 | **2.02** | **(1.04-3.92)** |  | 71 | 54 | 1.56 | (0.95-2.57) | 23 | 18 | 1.20 | (0.46-3.14) |  |
| p-trend |  |  |  | 0.89 |  |  |  | **0.04** | 0.24 |  |  |  | **0.04** |  |  |  | 0.83 | 0.19 |
|  |  |  |  |  |  |  |  |  |  |  |  |  |  |  |  |  |  |  |
| *trans*-Nonachlor |  |  |  |  |  |  |  |  |  |  |  |  |  |  |  |  |  |  |
| <LOD | 35 | 43 | 1.00 | Ref. | 148 | 166 | 1.00 | Ref. |  | 121 | 135 | 1.00 | Ref. | 26 | 27 | 1.00 | Ref. |  |
| ≥LOD-8.92 | 34 | 44 | 0.80 | (0.40-1.57) | 81 | 88 | 1.02 | (0.69-1.52) |  | 68 | 92 | 0.84 | (0.55-1.27) | 24 | 21 | 1.19 | (0.51-2.79) |  |
| 8.93-15.84 | 76 | 78 | 0.98 | (0.54-1.79) | 65 | 53 | 1.55 | (0.98-2.48) |  | 94 | 83 | 1.29 | (0.85-1.96) | 23 | 27 | 0.90 | (0.34-2.38) |  |
| 15.85-89.20 | 106 | 86 | 1.28 | (0.68-2.39) | 53 | 42 | 1.39 | (0.82-2.38) |  | 101 | 75 | 1.46 | (0.92-2.31) | 27 | 26 | 0.95 | (0.34-2.63) |  |
| p-trend |  |  |  | 0.20 |  |  |  | 0.22 | 0.72 |  |  |  | **0.06** |  |  |  | 0.94 |  |
|  |  |  |  |  |  |  |  |  |  |  |  |  |  |  |  |  |  |  |
| β-HCCH |  |  |  |  |  |  |  |  |  |  |  |  |  |  |  |  |  | 0.44 |
| <LOD | 140 | 162 | 1.00 | Ref. | 308 | 315 | 1.00 | Ref. |  | 297 | 314 | 1.00 | Ref. | 70 | 80 | 1.00 | Ref. |  |
| ≥LOD (2.77-153.80) | 107 | 85 | 1.27 | (0.84-1.92) | 35 | 30 | 1.05 | (0.61-1.79) | 0.54 | 82 | 68 | 1.14 | (0.75-1.72) | 29 | 19 | 1.70 | (0.71-4.08) | 0.41 |
|  |  |  |  |  |  |  |  |  |  |  |  |  |  |  |  |  |  |  |
| Mirex |  |  |  |  |  |  |  |  |  |  |  |  |  |  |  |  |  |  |
| <LOD | 186 | 179 | 1.00 | Ref. | 319 | 323 | 1.00 | Ref. |  | 330 | 337 | 1.00 | Ref. | 73 | 67 | 1.00 | Ref. |  |
| ≥LOD (2.88-348.20) | 67 | 70 | 0.96 | (0.63-1.48) | 21 | 20 | 1.02 | (0.51-2.06) | 0.78 | 51 | 47 | 1.10 | (0.69-1.74) | 25 | 31 | 0.52 | (0.23-1.19) | 0.15 |
| All models adjusted for BMI (<25 kg/m2, 25-29.9 kg/m2, ≥30 kg/m^2^, missing) and military branch (Army, Air Force, Navy, Marines/Coast Guard) | | | | | | | | | | | | | | | | | | |
| Abbreviations: CI = confidence interval; DDE = dichlorodiphenyldichloroethylene; DDT = dichlorodiphenyltrichloroethane; HCB = hexachlorobenzene; HCCH = hexachlorocyclohexane; LOD = limit of detection; OR = odds ratio | | | | | | | | | | | | | | | | | | |

| Supplemental Table 2. Associations between lipid-corrected serum concentrations of organochlorine pesticides and risk of classical papillary thyroid cancer, restricted to cases and matched controls with serum samples collected five or more years prior to diagnosis | | | | | | | | | | | | | | |
| --- | --- | --- | --- | --- | --- | --- | --- | --- | --- | --- | --- | --- | --- | --- |
| OCP levels (ng/g lipid) | Overall (N=627 case-control pairs) | | | |  | Restricted to classical PTC (N=508 case-control pairs) | | | |  | Restricted to classical PTC and females only (N=222 case-control pairs) | | | |
|  | N_cases_ | N_controls_ | OR | 95% CI |  | N_cases_ | N_controls_ | OR | 95% CI |  | N_cases_ | N_controls_ | OR | 95% CI |
| *p,p'*-DDE |  |  |  |  |  |  |  |  |  |  |  |  |  |  |
| <LOD-78.56 | 132 | 140 | 1.00 | Ref. |  | 106 | 112 | 1.00 | Ref. |  | 52 | 55 | 1.00 | Ref. |
| 78.57-129.69 | 156 | 159 | 0.97 | (0.68-1.37) |  | 121 | 130 | 0.94 | (0.63-1.40) |  | 47 | 59 | 0.90 | (0.49-1.64) |
| 129.70-233.49 | 179 | 166 | 1.06 | (0.74-1.52) |  | 149 | 134 | 1.11 | (0.74-1.66) |  | 63 | 49 | 1.45 | (0.78-2.71) |
| 233.50-2,049.00 | 160 | 162 | 0.91 | (0.60-1.37) |  | 132 | 132 | 0.95 | (0.60-1.52) |  | 60 | 59 | 1.20 | (0.59-2.44) |
| p-trend |  |  |  | 0.86 |  |  |  |  | 0.83 |  |  |  |  | 0.63 |
|  |  |  |  |  |  |  |  |  |  |  |  |  |  |  |
| *p,p'*-DDT |  |  |  |  |  |  |  |  |  |  |  |  |  |  |
| <LOD | 349 | 358 | 1.00 | Ref. |  | 275 | 296 | 1.00 | Ref. |  | 122 | 132 | 1.00 | Ref. |
| ≥LOD-6.30 | 75 | 88 | 0.86 | (0.61-1.22) |  | 62 | 71 | 0.93 | (0.63-1.36) |  | 19 | 26 | 0.87 | (0.45-1.69) |
| 6.31-10.35 | 100 | 87 | 1.10 | (0.75-1.60) |  | 90 | 72 | 1.28 | (0.85-1.93) |  | 46 | 33 | 1.63 | (0.89-2.98) |
| 10.36-126.80 | 96 | 86 | 1.05 | (0.72-1.53) |  | 78 | 64 | 1.31 | (0.85-2.01) |  | 33 | 27 | 1.49 | (0.75-2.94) |
| p-trend |  |  |  | 0.89 |  |  |  |  | 0.82 |  |  |  |  | 0.67 |
|  |  |  |  |  |  |  |  |  |  |  |  |  |  |  |
| HCB |  |  |  |  |  |  |  |  |  |  |  |  |  |  |
| <LOD | 301 | 311 | 1.00 | Ref. |  | 239 | 258 | 1.00 | Ref. |  | 120 | 108 | 1.00 | Ref. |
| ≥LOD-6.36 | 104 | 99 | 1.05 | (0.75-1.48) |  | 87 | 86 | 1.09 | (0.75-1.57) |  | 26 | 31 | 1.47 | (0.81-2.65) |
| 6.37-8.64 | 90 | 98 | 0.89 | (0.62-1.28) |  | 73 | 73 | 1.09 | (0.72-1.63) |  | 35 | 29 | 1.06 | (0.54-2.06) |
| 8.65-150.70 | 118 | 100 | 1.13 | (0.77-1.65) |  | 101 | 76 | 1.50 | (0.98-2.29) |  | 134 | 50 | **1.95** | **(1.01-3.79)** |
| p-trend |  |  |  | 0.65 |  |  |  |  | 0.18 |  |  |  |  | 0.13 |
|  |  |  |  |  |  |  |  |  |  |  |  |  |  |  |
| Oxychlordane |  |  |  |  |  |  |  |  |  |  |  |  |  |  |
| <LOD | 269 | 298 | 1.00 | Ref. |  | 214 | 243 | 1.00 | Ref. |  | 102 | 113 | 1.00 | Ref. |
| ≥LOD-7.95 | 111 | 108 | 1.11 | (0.79-1.57) |  | 91 | 90 | 1.16 | (0.80-1.68) |  | 34 | 35 | 1.15 | (0.64-2.06) |
| 7.96-12.65 | 126 | 109 | 1.23 | (0.87-1.75) |  | 105 | 89 | 1.32 | (0.89-1.94) |  | 45 | 41 | 1.37 | (0.75-2.52) |
| 12.66-104.80 | 119 | 109 | 1.16 | (0.80-1.68) |  | 96 | 83 | 1.28 | (0.85-1.93) |  | 40 | 31 | 1.57 | (0.83-2.97) |
| p-trend |  |  |  | 0.42 |  |  |  |  | 0.29 |  |  |  |  | 0.25 |
|  |  |  |  |  |  |  |  |  |  |  |  |  |  |  |
| *trans*-Nonachlor |  |  |  |  |  |  |  |  |  |  |  |  |  |  |
| <LOD | 181 | 196 | 1.00 | Ref. |  | 146 | 159 | 1.00 | Ref. |  | 76 | 75 | 1.00 | Ref. |
| ≥LOD-8.92 | 123 | 141 | 0.93 | (0.66-1.30) |  | 92 | 117 | 0.84 | (0.58-1.21) |  | 41 | 50 | 0.80 | (0.46-1.40) |
| 8.93-15.84 | 149 | 144 | 1.15 | (0.81-1.64) |  | 129 | 117 | 1.25 | (0.86-1.82) |  | 52 | 55 | 0.96 | (0.54-1.71) |
| 15.85-304.30 | 172 | 146 | 1.29 | (0.89-1.87) |  | 139 | 115 | 1.33 | (0.88-2.00) |  | 51 | 42 | 1.22 | (0.64-2.32) |
| p-trend |  |  |  | 0.20 |  |  |  |  | 0.20 |  |  |  |  | 0.58 |
| β-HCCH |  |  |  |  |  |  |  |  |  |  |  |  |  |  |
| <LOD | 471 | 496 | 1.00 | Ref. |  | 374 | 399 | 1.00 | Ref. |  | 159 | 181 | 1.00 | Ref. |
| ≥LOD (2.77-153.80) | 143 | 124 | 1.11 | (0.82-1.50) |  | 125 | 102 | 1.20 | (0.86-1.67) |  | 59 | 40 | **1.82** | **(1.07-3.08)** |
|  |  |  |  |  |  |  |  |  |  |  |  |  |  |  |
| Mirex |  |  |  |  |  |  |  |  |  |  |  |  |  |  |
| <LOD | 522 | 521 | 1.00 | Ref. |  | 425 | 424 | 1.00 | Ref. |  | 191 | 191 | 1.00 | Ref. |
| ≥LOD (2.88-348.20) | 99 | 98 | 1.05 | (0.75-1.47) |  | 80 | 79 | 1.03 | (0.70-1.50) |  | 29 | 27 | 1.10 | (0.59-2.06) |
| All models adjusted for BMI (<25 kg/m2, 25-29.9 kg/m2, ≥30 kg/m^2^, missing) and military branch (Army, Air Force, Navy, Marines/Coast Guard) | | | | | | | | | | | | | | |
| Bolding denotes statistical significance | | | | | | | | | | | | | | |
| Abbreviations: CI = confidence interval; DDE = dichlorodiphenyldichloroethylene; DDT = dichlorodiphenyltrichloroethane; HCB = hexachlorobenzene; HCCH = hexachlorocyclohexane; LOD = limit of detection; OR = odds ratio | | | | | | | | | | | | | | |
